# Histopathological Images and Multi-Omics Integration Predict Molecular Characteristics and Survival in Lung Adenocarcinoma

**DOI:** 10.3389/fcell.2021.720110

**Published:** 2021-10-11

**Authors:** Linyan Chen, Hao Zeng, Yu Xiang, Yeqian Huang, Yuling Luo, Xuelei Ma

**Affiliations:** ^1^State Key Laboratory of Biotherapy, Department of Biotherapy, Cancer Center, West China Hospital, Sichuan University, Chengdu, China; ^2^Department of Pathology, West China Hospital, Sichuan University, Chengdu, China

**Keywords:** lung cancer, histopathology, genomics, transcriptomics, proteomics

## Abstract

Histopathological images and omics profiles play important roles in prognosis of cancer patients. Here, we extracted quantitative features from histopathological images to predict molecular characteristics and prognosis, and integrated image features with mutations, transcriptomics, and proteomics data for prognosis prediction in lung adenocarcinoma (LUAD). Patients obtained from The Cancer Genome Atlas (TCGA) were divided into training set (*n* = 235) and test set (*n* = 235). We developed machine learning models in training set and estimated their predictive performance in test set. In test set, the machine learning models could predict genetic aberrations: *ALK* (AUC = 0.879), *BRAF* (AUC = 0.847), *EGFR* (AUC = 0.855), *ROS1* (AUC = 0.848), and transcriptional subtypes: proximal-inflammatory (AUC = 0.897), proximal-proliferative (AUC = 0.861), and terminal respiratory unit (AUC = 0.894) from histopathological images. Moreover, we obtained tissue microarrays from 316 LUAD patients, including four external validation sets. The prognostic model using image features was predictive of overall survival in test and four validation sets, with 5-year AUCs from 0.717 to 0.825. High-risk and low-risk groups stratified by the model showed different survival in test set (HR = 4.94, *p* < 0.0001) and three validation sets (HR = 1.64–2.20, *p* < 0.05). The combination of image features and single omics had greater prognostic power in test set, such as histopathology + transcriptomics model (5-year AUC = 0.840; HR = 7.34, *p* < 0.0001). Finally, the model integrating image features with multi-omics achieved the best performance (5-year AUC = 0.908; HR = 19.98, *p* < 0.0001). Our results indicated that the machine learning models based on histopathological image features could predict genetic aberrations, transcriptional subtypes, and survival outcomes of LUAD patients. The integration of histopathological images and multi-omics may provide better survival prediction for LUAD.

## Introduction

Lung cancer is the most common cancer and the main cause of cancer death worldwide, resulting in an estimated 2.1 million new cases and 1.8 million deaths annually (Bray et al., 2018). Lung adenocarcinoma (LUAD) is the most major histological subtype, which is different from lung squamous cell carcinoma (LUSC) in clinical manifestations and therapeutic principles (Ettinger et al., 2019). LUAD occurs more frequently in never-smokers compared to LUSC (Herbst et al., 2018). Although there were small improvements in 5-year survival rate of lung cancer patients, the survival rates of patients with lymph node invasion (29.7%) or distant metastases (4.7%) were still not optimistic (Schabath and Cote, 2019). Therefore, identifying high-risk patients with worse prognosis is critical to the treatment and management of cancer patients. In recent years, various novel biomarkers are constantly emerging to better classify LUAD patients by their probable prognosis, and promote the development of precision medicine (Vargas and Harris, 2016).

Oncogene addiction refers to the dependence of cancer cells on specific driver oncogenes that play important roles in tumorigenesis and progression (Pagliarini et al., 2015). Known driver oncogene aberrations of LUAD included mutations of *KRAS*, *EGFR*, and *BRAF* genes, and fusions of *ALK*, *ROS1*, and *RET* genes (Saito et al., 2016). Based on the concept of “oncogene addiction,” cancer patients harboring such aberrations can benefit from molecular targeted therapy. Targeted agents against lung cancers with *EGFR*, *BRAF*, *ALK*, and *ROS1* aberrations (e.g., gefitinib, dabrafenib, and crizotinib) have achieved good efficacy in clinical trials, which are successful examples of clinical application of precision therapy (Saito et al., 2016; Park et al., 2019). The *EGFR* and *BRAF* mutations were found to be correlated with patient prognosis, but their prognostic implication in LUAD remains controversial (Calvayrac et al., 2017). In addition, based on unsupervised analysis of gene expression, the transcriptional subtypes of LUAD were proposed to provide clinical related classifications, and offered insights into tumors that lacked specific driver mutations (Cancer Genome Atlas Research Network, 2014). The terminal respiratory unit (TRU), proximal-inflammatory (PI), and proximal-proliferative (PP) subtypes revealed distinct genomic signatures, and the TRU subtype had a favorable prognosis (Cancer Genome Atlas Research Network, 2014). Furthermore, proteomic characterization of LUAD demonstrated the association of proteomic features with genetic aberrations and transcriptional subtypes (Gillette et al., 2020). Overall, multi-omics analysis is crucial for understanding the molecular landscape of cancer and improving the prognosis prediction and therapeutic strategies of patients.

Several clinicopathological factors are well-recognized prognostic factors for LUAD, such as TNM stage, smoking history, and performance status (Thakur and Gadgeel, 2016). Histopathological images contain numerous information about tumor morphology and its correlation with surrounding microenvironment. However, current histopathological assessment patterns (such as classification of tumor grade) are not sufficient to predict prognosis of LUAD patients (Yu et al., 2016). Computer-aided image analysis is an emerging field of artificial intelligence that converts digital pathological images into high-dimensional data, and offers a new approach to studying tumor heterogeneity and underlying pathophysiological mechanisms (Zhang et al., 2015). This digital innovation has potential to promote the modernization of pathology workflow, improve efficiency and consistency while maintaining diagnostic and prognostic accuracy, and provide decision support for clinicians (Niazi et al., 2019). Various types of quantitative image features have been defined, such as the cell size, shape, spatial distribution, and texture patterns (Tabesh et al., 2007). Based on histopathological image features, machine learning models have shown its utility in predicting tumor classification and patient outcome, such as lung (Yu et al., 2016), breast (Turkki et al., 2019), and prostate cancers (Lee et al., 2017). Furthermore, previous study indicated that the prognostic performance of histopathological image features was independent of other clinical factors in LUAD, including age, gender, tumor stage, and smoking status (Luo et al., 2017). Considering the properties of tumor and its microenvironment are closely related to molecular alterations, many researches have been conducted on the genomics and histopathological features. For example, promising result for the prediction of commonly mutated genes in lung cancer from histopathological images was reported (Coudray et al., 2018). Recent studies suggested that the integration of histopathological images and genomics data can enhance the ability to predict survival of cancer patients compared with using only one type of data (Cheng et al., 2017; Mobadersany et al., 2018; Zeng et al., 2020).

In this article, we designed an image processing pipeline to automatically extract image features from digital histopathological slides, and performed systematic analyses to correlate the features from histopathological images and omics profiles. Firstly, besides common mutations, we also built machine learning classifiers to predict the transcriptional subtypes of LUAD. In addition, we used histopathological image features alone or integrated them with genomics, transcriptomics and proteomics data to establish predictive models, and evaluated their prognostic roles for patient survival in independent datasets. We expected that the integrative models would more reliably predict survival risk and contribute to the personalized medicine of LUAD patients.

## Materials and Methods

### Patient Cohorts

This study included two independent data sources. Firstly, hematoxylin and eosin (H&E)-stained histopathological images of 522 LUAD patients were obtained from The Cancer Imaging Archive (TCIA),^1^ whereas the corresponding genomics, transcriptomics, and proteomics information were downloaded from The Cancer Genome Atlas (TCGA)^2^ and The Cancer Proteome Atlas (TCPA) repositories.^3^ The inclusion criteria were surgically resected LUAD patients with available histopathological images, genomics, and transcriptomics data, and 470 patients were finally included in this study. In addition, tissue microarrays (TMAs) of 316 LUAD patients were acquired from Shanghai Outdo Biotech Company (Shanghai, China). The TMA-LUAD datasets contained four cohorts of patients (HLugA150Su01, *n* = 69; HLugA150Su02, *n* = 72; HLugA180Su04, *n* = 86; HLugA180Su05, *n* = 89). All tumor samples were collected by surgical resection. The pathologists first labeled the representative areas in formalin-fixed and paraffin-embedded tissues. The labeled tissues were arranged on the blank paraffin-embedded blocks by semi-automated tissue arrayer (TMArrayer), and the blocks were continuously sliced into TMAs. Digital TMA images were scanned by Aperio AT2 slide scanner. The utilization of TMAs was approved by the National Human Genetic Resources Sharing Service Platform (2005DKA21300), and informed consent was obtained from all patients.

### Image Segmentation and Feature Extraction

**FIGURE 1:**
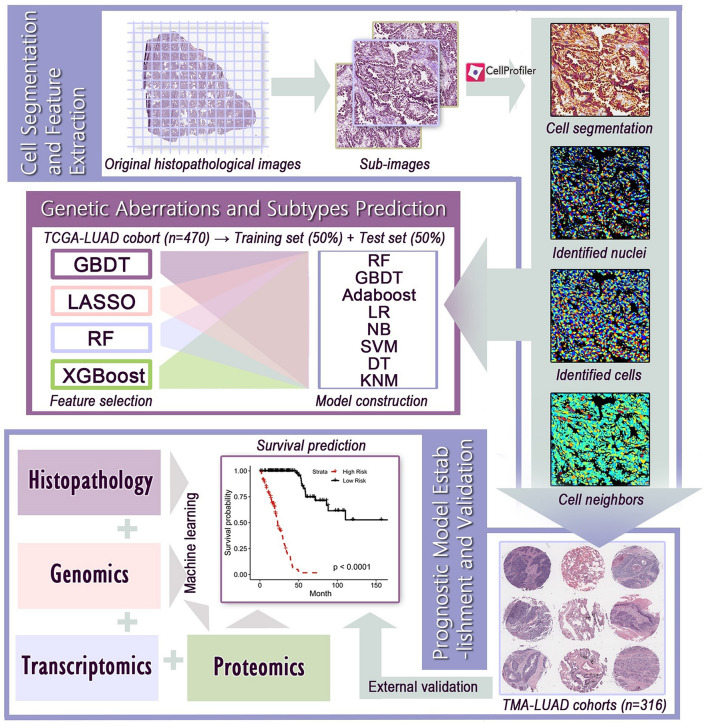
The flowchart of histopathological image processing and data integration. (1) Whole-slide images were cropped into sub-images of 1,000 × 1,000 pixels, and 60 sub-images were randomly selected. CellProfiler was used to identify the nuclei and cells, and extract histopathological image features. (2) Image features and various machine learning algorithms were used to predict genetic aberrations and transcriptional subtypes in training and test sets. (3) Prognostic models were established using image features alone or integration of image features, genomics, transcriptomics, and proteomics, and the prognostic value was evaluated in test set or validation sets.

The workflow of image processing and data integration was outlined in Figure 1. Since the original histopathological images (40× magnification) had extreme high resolution, we first cropped whole-slide images into millions of sub-images of 1,000 × 1,000 pixels through the Openslide-Python (Goode et al., 2013), and 60 sub-images from each whole-slide image for further analysis. The cell morphology of randomly selected sub-images still had good consistency (Supplementary Figure 1). Because the TMAs had much smaller size than whole-slide images, we applied the same processing method with adjusted size constraint to crop TMAs, and used all sub-images for feature extraction. We next used CellProfiler (McQuin et al., 2018) to separate the hematoxylin and eosin stains of sub-images and corrected the illumination. Then, CellProfiler automatically segmented the nuclei and cells through “Identify Primary/Secondary Objects” modules, and extracted specific image features from these cellular regions through 10 measurement modules, such as “Measure Object Size Shape,” “Measure Correlation,” “Measure Object Neighbors,” and “Measure Texture” modules. These image features quantified the cell-level morphological characteristics, texture properties, and the relationship between neighboring objects. A total of 536 histopathological image features listed in Supplementary Table 2 were included in this study.

### Genetic Aberrations and Transcriptional Subtype Prediction

The TCGA cohort was randomly assigned into training set and test set in proportion of 1:1. In the training set, we first used gradient boosting decision tree (GBDT) (Friedman, 2001), least absolute shrinkage and selection operator (LASSO) (Tibshirani, 1997), random forest (RF) (Breiman, 2001), and extreme gradient boosting (XGBoost) (Chen and Guestrin, 2016) to select relevant histopathological image features to reduce the risk of over-fitting. We used GBDT, RF, or XGBoost to select the top 15 features based on the ranking of importance. LASSO automatically shrunk the regression coefficients of irrelevant features to zero, and remained a variable number of features with non-zero coefficients for building classifier. Next, RF, GBDT, adaptive boosting (AdaBoost) (Collins et al., 2002), logistic regression (LR) (Collins et al., 2002), naive Bayes (NB) (Rish, 2001), support vector machine (SVM) (Cortes and Vapnik, 1995), decision tree (DT) (Safavian and Landgrebe, 1991), and K-nearest neighbor (KNN) (Weinberger and Saul, 2009) were applied to build models based on the selected features to predict genetic aberrations (*ALK*, *BRAF*, *EGFR*, *ROS1*) and transcriptional subtypes (PI, PP, TRU). We performed fivefold cross-validation on the training set to determine optimal feature combination and ensure the robustness of models. After feature selection and model construction on the training set, the predictive power of models was evaluated by the area under curve (AUC) of receiver operating characteristic (ROC) curve on the test set. True positive rate (TPR, sensitivity) refers to the proportion of predicted positive samples in all true positive samples. False positive rate (FPR, 1-specificity) is the proportion of samples that are incorrectly predicted as positive in all true negative samples.

### Prognostic Model Establishment and Validation

1. Prognostic analysis of histopathological image features: By the median value of each feature, patients of training set were separated into high-value group and low-value group. Then, we conducted Cox regression analysis to estimate the prognostic effect of individual features on overall survival (OS), and calculated the hazard ratio (HR) and 95% confidence interval (CI). Kaplan-Meier survival curves and log-rank test estimated the survival outcomes of two groups. Prognostic value was significant if *p* < 0.05.

2. Data processing: Before modeling, we screened features of genomics and transcriptomics to reduce data dimension, while using all proteomics and histopathological image features. In the training set, the top 100 somatic mutations were included. To identity a limited subset of expressed genes from the whole transcriptomics profile, we selected the 100 most differently expressed genes (DEGs) between short-term (OS of 1–12 months at death) and long-term (OS ≥ 60 months) patients of training set in our models. DEGs between groups were identified by R package ‘‘DESeq2.’’ Based on DEGs, we also analyzed the enriched gene function by Gene Ontology (GO) enrichment analysis on Metascape.^4^

3. Model development: We designed multiple data integration to build prognostic models, including one type of features (histopathological images, genomics, transcriptomics, proteomics), combinations of two types of features (histopathology + genomics, histopathology + transcriptomics, histopathology + proteomics), and all features (multi-omics). Input features were selected to establish models for predicting prognosis in the training set through RF (R package “randomForestSRC”) with fivefold cross-validation. Survival risk for each patient was assessed by models, then patients were divided into high-risk and low-risk groups according to the median risk score. Time-dependent ROC curve, Kaplan-Meier method, and log-rank test evaluated the predictive capability, while decision curve analysis compared the clinical net benefit of each model. We further estimated the performance of these models in the test set. The model based on histopathological images was externally validated by the TMA-LUAD cohorts. Statistical analyses were performed using R 3.6.1.

## Results

### Prediction of Genetic Aberrations and Transcriptional Subtypes

To assess whether machine learning can be trained to predict genetic aberrations and transcriptional subtypes using histopathological image features as input, we downloaded the related data from TCGA. The TCGA-LUAD cohort was then randomly divided into training set (*n* = 235) and test set (*n* = 235). The baseline clinical and molecular characteristics were not significantly different between the two sets (Table 1). To compare the feasibility of various machine learning methods, we applied four algorithms (GBDT, LASSO, RF, XGBoost) to select features and eight algorithms (RF, GBDT, AdaBoost, LR, NB, SVM, DT, KNN) to build models in the training set, which generated 32 combinations of two algorithms. The predictive performance of these models was estimated in the test set.

**TABLE 1:** Patient characteristics of the TCGA cohort.

**Characteristics**	**TCGA-LUAD**		**p**
	**Training set (n = 235)**	**Test set (*n* = 235)**	
Age: mean ± SD	65.1 ± 9.5	65.4 ± 10.5	0.740
**Gender (%)**			
Male	114 (48.5)	106 (45.1)	
Female	121 (51.5)	129 (54.9)	0.460
**Tumor stage (%)**			
I	132 (56.2)	122 (51.9)	
II	55 (23.4)	60 (25.5)	
III	34 (14.5)	42 (17.9)	
IV	13 (5.5)	10 (4.3)	
NA	1 (0.4)	1 (0.4)	0.605
**Smoking status (%)**			
Non-smoker	29 (12.3)	33 (14.0)	
Current smoker	53 (22.6)	56 (23.9)	
Former smoker	147 (63.6)	139 (59.1)	
NA	6 (2.6)	7 (3.0)	0.755
**Survival status (%)**			
Alive	141 (60.0)	160 (68.1)	
Deceased	94 (40.0)	75 (31.9)	0.068
***ALK* translocation (%)**			
−	106 (45.1)	91 (38.7)	
+	18 (7.7)	13 (5.5)	
NA	111 (47.2)	131 (55.7)	0.658
***BRAF* mutation (%)**			
−	219 (93.2)	225 (95.7)	
+	16 (6.8)	10 (4.3)	0.226
***EGFR* mutation (%)**			
−	210 (89.4)	200 (85.1)	
+	25 (10.6)	35 (14.9)	0.167
***ROS1* translocation (%)**			
−	228 (97.0)	219 (93.2)	
+	7 (3.0)	16 (6.8)	0.054
**Transcriptional subtype (%)**			
Proximal-inflammatory	32 (13.6)	39 (16.6)	
Proximal-proliferative	25 (10.6)	32 (13.6)	
Terminal respiratory unit	48 (20.4)	33 (14.0)	
NA	130 (55.3)	131 (55.7)	0.115

**FIGURE 2:**
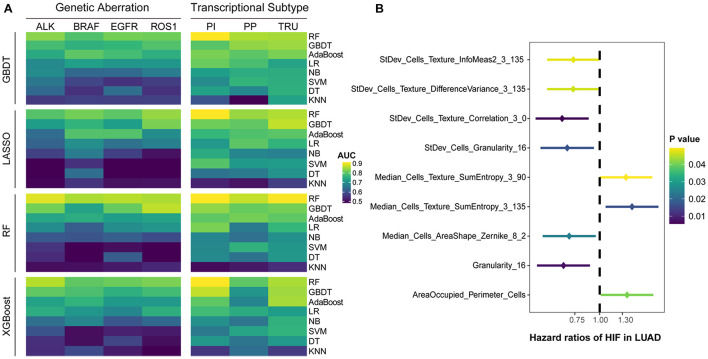
Predictive performance of histopathological image features. **(A)** Four algorithms (GBDT, LASSO, RF, XGBoost) were utilized to select image features, and eight algorithms (RF, GBDT, AdaBoost, LR, DT, SVM, NB, KNN) were used for modeling in training set. The ability of models to predict genetic aberrations and transcriptional subtypes was assessed in test set. **(B)** Histopathological image features (HIF) with significant prognostic value (*p* < 0.05) in univariate Cox analysis.

The results showed that the predictive models constructed by RF had the highest accuracy on the test set, regardless of the type of feature selection algorithm employed (Figure 2A). The models built only by RF were capable of predicting common gene aberrations in LUAD: *ALK* (AUC = 0.879), *BRAF* (AUC = 0.847), *EGFR* (AUC = 0.855), *ROS1* (AUC = 0.848), and transcriptional subtypes: PI (AUC = 0.897), PP (AUC = 0.861), and TRU (AUC = 0.894) (Supplementary Table 1). The combination of XGBoost and RF, XGBoost and GBDT, and GBDT and RF also performed well on the test set (Supplementary Table 1). Our analyses suggested that the genetic aberrations and transcriptional subtypes of LUAD could be predicted by histopathological image features with machine learning.

### Evaluation and Validation of Image Features to Predict Survival

We assigned the training set into two groups by the median value of each histopathological image feature, and performed univariate Cox analysis to evaluate their prognostic value of overall survival (OS) (Supplementary Table 2). The results demonstrated that nine features may be able to predict OS (Figure 2B). With most of these features, the high-value group displayed a longer survival in Kaplan-Meier survival curve (Supplementary Figure 2), such as the four features with the lowest *p*-values: StDev_Cells_Texture_Correlation_3_0 (HR=0.65, 95% CI: 0.48–0.88, *p* = 0.006), Granularity_16 (HR=0.66, 95% CI: 0.48–0.89, *p* = 0.007), StDev_Cells_Granularity_16 (HR=0.69, 95% CI: 0.50–0.94, *p* = 0.017), and Median_Cells_AreaShape_Zernike_8_2 (HR=0.70, 95% CI: 0.52–0.96, *p* = 0.024).

**FIGURE 3:**
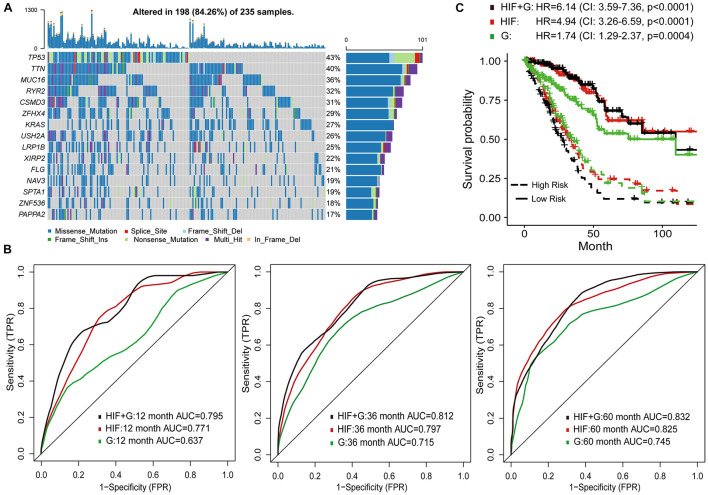
Prognostic models based on histopathological image features (HIF) and genomics (G). **(A)** The 15 most common somatic mutations in training set. **(B)** Predictive power of HIF model, genomics model, and HIF+G model for 1-, 3-, and 5-year survival in test set. **(C)** Kaplan-Meier survival curves of high-risk and low-risk groups of test set predicted by the three models.

To improve survival prediction with histopathological image features, we adopted random forest algorithm to select informative features from all image features and build prognostic model in the training set. We assessed the time-dependent ROC curves of the model in the test set (Figure 3B), which achieved good performance in predicting 1-year (AUC = 0.711), 3-year (AUC = 0.797), and 5-year OS (AUC = 0.825). Survival risk score calculated from the model divided patients into high-risk group (greater than median) and low-risk group (less than median). Compared with the individual features (Supplementary Figure 2), our model could better distinguish high-risk group from low-risk group in the test set (Figure 3C). High-risk patients had significant association with worse survival (HR = 4.94, 95% CI: 3.26–6.59, *p* < 0.0001).

**FIGURE 4:**
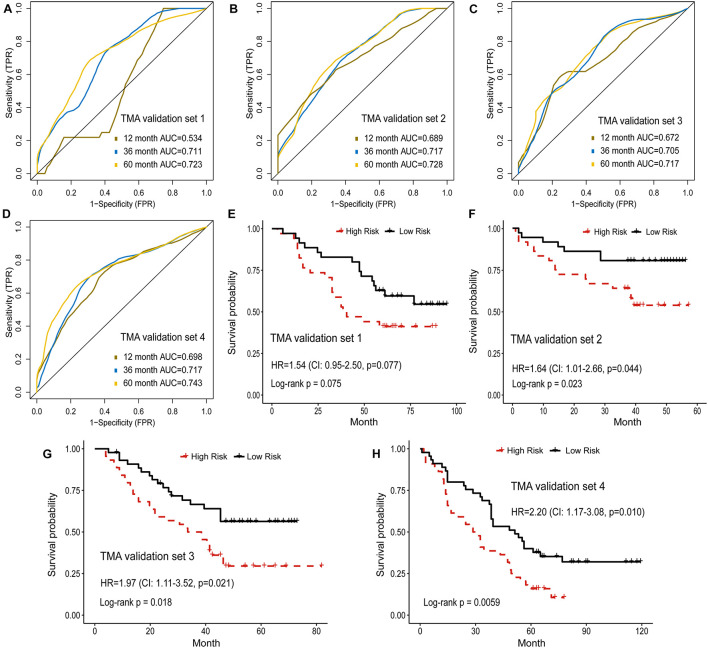
Validation of histopathological image features for survival prediction. **(A–D)** The 1-, 3-, and 5-year time-dependent ROC curves of the model built by histopathological image features in four validation sets. **(E–H)** Kaplan-Meier survival curves of validation sets analyzed with the image feature model.

In addition, the prognostic model based on image features was externally validated with the TMA-LUAD cohorts (Table 2). The AUC of predicting 5-year OS was 0.723 in validation set 1, 0.728 in validation set 2, 0.717 in validation set 3, and 0.743 in validation set 4 (Figures 4A–D). Although higher risk score was expected to be related to worse survival, this association was not strong in validation set 1 (*p* = 0.077; Figure 4E). However, there were significant survival differences in validation set 2 (HR = 1.64, 95% CI: 1.01–2.66, p = 0.044), validation set 3 (HR = 1.97, 95% CI: 1.11–3.52, *p* = 0.021), and validation set 4 (HR = 2.20, 95% CI: 1.17–3.08, *p* = 0.010; Figures 4F–H). Supplementary Figure 3 presented several examples of histopathological images of high-risk and low-risk groups in the TCGA and TMA cohorts. In the TMA validation sets, patients with the same histological grade had different survival results. The subtle morphological differences between tumor cells of the same grade were not easily distinguished by visual evaluation, but could be quantified through cell segmentation and feature extraction on images. These results indicated the feasibility of histopathological image features for survival prediction in LUAD patients.

**TABLE 2:** Patient characteristics of the TMA cohorts.

**Characteristics**	**TMA-LUAD**			
	**Validation set 1**	**Validation set 2**	**Validation set 3**	**Validation set 4**
No. of patients	69	72	86	89
Microarray number	HLugA150Su01	HLugA150Su02	HLugA180Su04	HLugA180Su05
Surgery time	2004.7–2007.8	2007.9–2009.6	2008.1–2013.7	2004.7–2009.6
Last follow-up	2012.7	2012.7	2016.6	2014.8
Age: mean ± SD	58.6 ± 12.2	59.7 ± 10.7	62.8 ± 10.0	62.5 ± 9.9
**Gender (%)**				
Male	35 (50.7)	39 (54.2)	47 (54.7)	48 (53.9)
Female	34 (49.3)	33 (45.8)	39 (45.3)	41 (46.1)
**Tumor stage (%)**				
I	30 (43.5)	23 (31.9)	23 (26.7)	27 (30.3)
II	10 (14.5)	14 (19.4)	14 (16.3)	16 (18.0)
III	16 (23.2)	18 (25.0)	18 (20.9)	28 (31.5)
IV	3 (4.3)	0 (0.0)	2 (2.3)	1 (1.1)
NA	10 (14.5)	17 (23.6)	29 (33.7)	17 (19.1)
**Histological grade (%)**				
G1	10 (14.5)	6 (8.3)	0 (0.0)	3 (3.4)
G1-2	3 (4.3)	5 (6.9)	9 (10.5)	4 (4.5)
G2	37 (53.6)	45 (62.5)	48 (55.8)	55 (61.8)
G2-3	9 (13.0)	3 (4.2)	23 (26.7)	16 (18.0)
G3	10 (14.5)	13 (18.1)	6 (7.0)	11 (12.4)
**Survival status (%)**				
Alive	34 (49.3)	49 (68.1)	38 (44.2)	21 (23.6)
Deceased	35 (50.7)	23 (31.9)	48 (55.8)	68 (76.4)

### Integration of Image Features and Genomics for Survival Prediction

Genetic aberrations especially driver oncogenes were closely related to the genesis and development of tumors. Here, we estimated the prognostic role of genomics data, and attempted to integrate image features with genomics to enhance survival prediction. We selected 100 genes with the highest mutation frequency in the training set for modeling (Supplementary Table 3 and Figure 3A). The process of data analysis and random forest method were the same as above. As shown in Figure 3B, the prognostic model of histopathological image features (HIF) performed better than the genomics model (G) on the test set. Integration of image features and genomics (HIF + G) greatly improved the AUCs of 1-year (0.795 vs. 0.637), 3-year (0.812 vs. 0.715), and 5-year OS (0.832 vs. 0.745) compared to genomics alone, indicating the complementary value of image features in prognostic models. According to the risk score of each model, we further stratified the test set into high-risk and low-risk sub-sets, and found that the integrative model had significant predictive ability for OS (HR = 6.14, 95% CI: 3.59–7.36, *p* < 0.0001; Figure 3C).

### Integration of Image Features and Transcriptomics for Survival Prediction

**FIGURE 5:**
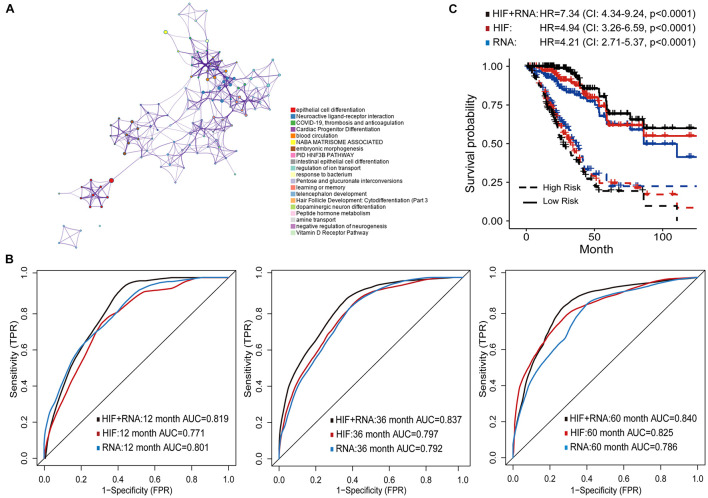
Prognostic models based on histopathological image features (HIF) and transcriptomics (RNA). **(A)** Gene Ontology enrichment network of differently expressed genes. The most enriched term was used to describe each cluster (see legends). **(B)** Area under the time-dependent ROC curves of HIF model, transcriptomics model, and HIF + RNA model in test set. **(C)** Kaplan-Meier survival curves of the three models in test set.

Besides genomics analysis, the transcriptomics profile has become increasingly important for understanding the tumor molecular characteristics. Therefore, we combined histopathological image features and mRNA transcription data to more accurately predict prognosis of LUAD patients. We analyzed the differently expressed genes (DEGs) between short-term (OS of 1–12 months at death, *n* = 24) and long-term (OS ≥ 60 months, *n* = 29) patients of training set. GO enrichment analysis was next conducted to show the regulatory functions of DEGs. The GO enrichment terms were most abundant in cell differentiation, such as the differentiation of epithelial cells, cardiac progenitors, and neurons. These genes were also associated with functions of thrombosis, anticoagulation, and blood circulation (Figure 5A).

Next, the top 100 DEGs were involved in the modeling process (Supplementary Table 4). In the test set, the transcriptomics model (RNA) reached higher prognostic accuracy of 1-year OS (AUC = 0.801), but lower accuracy of 3-year (AUC = 0.792), and 5-year OS (AUC = 0.786) than the histopathological image feature (HIF) model (Figure 5B). The integrative model of image features and transcriptomics (HIF + RNA) achieved superior prediction results (1-year AUC = 0.819, 3-year AUC = 0.837, 5-year AUC = 0.840) than the other two models. Based on the HIF + RNA model, there was remarkable difference in OS between high-risk and low-risk patients of the test set (HR = 7.34, 95% CI: 4.34–9.24, *p* < 0.0001; Figure 5C).

### Integration of Image Features and Proteomics for Survival Prediction

**FIGURE 6:**
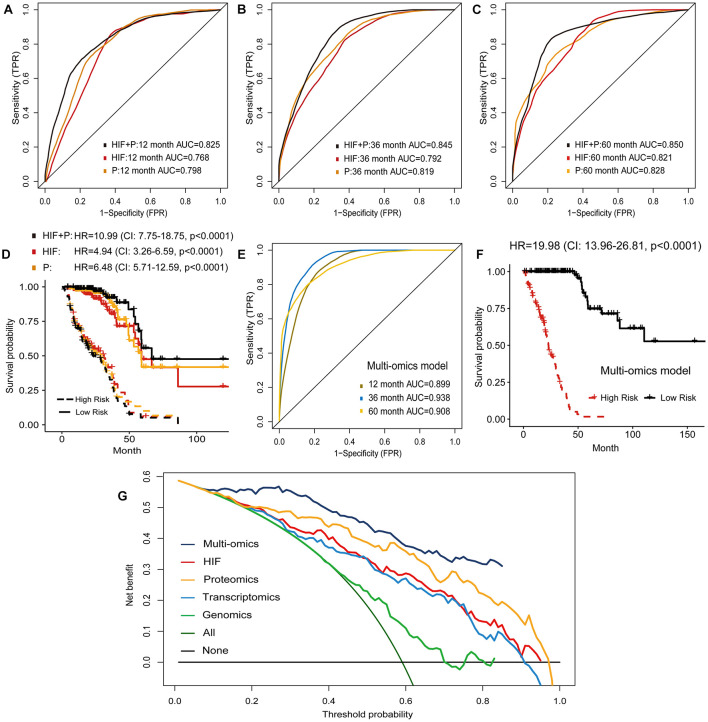
Prognostic models based on histopathological image features (HIF), proteomics (P), and multi-omics. **(A–C,E)** Predictive performance of HIF model, proteomics model, HIF + P model, and multi-omics model in test set. **(D,F)** Kaplan-Meier survival curves of high-risk and low-risk patients in test set based on these models. **(G)** Decision curve analysis. The horizontal black line was net benefit of treating no patient, and oblique green line was net benefit of treating all patients. The net benefit of treating patients according to the multi-omics model was highest when threshold probability was greater than 10%.

The proteomics characteristics can reflect the properties and mechanisms of tumor, and offer an opportunity for better prognosis prediction. Therefore, the expression levels of 218 proteins were used to establish the prognostic models (Supplementary Table 5). The proteomics model (P) performed comparably to the histopathological image feature model (HIF) in predicting survival of test set patients (Figures 6A–C). In addition, improved predictive performance was observed when the model was built by combining image features and proteomics data (HIF+P), which obtained AUC of 0.825, 0.845, and 0.850 for 1-, 3-, and 5-year OS, respectively. In the test set, the high-risk and low-risk patients predicted by these models displayed different survival outcomes (Figure 6D), especially the HIF + P model (HR = 10.99, 95% CI: 7.75–18.57, *p* < 0.0001).

### Integration of Image Features and Multiple Omics for Survival Prediction

The above results demonstrated that the combination of histopathological image features and single omics (genomics, transcriptomics, proteomics) provided a greater power to predict survival of LUAD patients. Accordingly, we investigated whether incorporating all omics and histopathological image features would cause further optimization. In the test set, the multi-omics model further enhanced 1-year AUC to 0.899, 3-year AUC to 0.938, and 5-year AUC to 0.908 for predicting OS (Figure 6E). Kaplan-Meier curves and Cox regression analysis showed that the predicted high-risk group of test set was remarkably related to worse prognosis (HR = 19.98, 95% CI: 13.96–26.81, *p* < 0.0001, Figure 6F). We also performed decision curve analysis of models on the test set, and the multi-omics model obtained higher net benefit than other models in clinical decision-making (Figure 6G).

## Discussion

Considering the importance of histopathological images in cancer prognosis, we proposed an image processing and data analysis workflow to extract quantitative features from histopathological images, and to develop predictive models for molecular characteristics and survival outcomes of LUAD patients using histopathological image features and machine learning methods. Moreover, we integrated image features and multiple omics information (i.e., somatic mutation, gene transcription, and protein expression) for survival prediction. From test analyses, the results showed that image features had significant value in predicting the genetic aberrations and transcriptional subtypes of LUAD. Survival prediction based on the integrative models became more precise than using image features or omics data alone, which indicated that image features and omics data may be complementary in prognosis prediction. The integrative models might better stratify high-risk and low-risk patients, which was beneficial for the treatment of LUAD patients.

The potential differences of molecular expression in tumors tend to present as the alterations of tissue structure and nuclear morphology (Madabhushi and Lee, 2016; Ni et al., 2021). Previously, it has been found that *STK11*, *EGFR*, *FAT1*, *SETBP1*, *KRAS*, and *TP53* mutations of LUAD could be predicted from histopathological images using deep learning (AUCs ranged from 0.733 to 0.856) (Coudray et al., 2018). Afterward, several studies also suggested that histopathological image analysis could detect the gene mutation status in liver, colorectal, and ovarian cancers (Chen et al., 2020; Jang et al., 2020; Zeng et al., 2021). In this study, although we also focused on the mutation prediction, we conducted multiple combinations of machine learning methods, and the random forest models had high prediction accuracy for *ALK* (AUC = 0.879), *BRAF* (AUC = 0.847), *EGFR* (AUC = 0.855), and *ROS1* (AUC = 0.848) aberrations. In our previous radiomics study of lung cancer, random forest also had good classification accuracy for primary and metastatic lung lesions (Zhou et al., 2021). Meanwhile, the models were trained to predict the transcriptional subtypes of LUAD for the first time, with AUCs from 0.861 to 0.897. The PP subtype had more *KRAS*-mutated and *STK11-*inactivated tumors. The PI subtype was enriched for solid histopathology, and co-mutation of *NF1* and *TP53*. Finally, the TRU subtype was characterized by *EGFR* mutation, kinase fusion expression, and better prognosis (Cancer Genome Atlas Research Network, 2014). Our finding indicated that gene mutation and expression may affect the patterns of tumor cells on histopathological images. Histopathological image features could be applied as a convenient and cost-effective approach to predict these important genetic aberrations and transcriptional subtypes in LUAD.

We evaluated the prognostic value of histopathological image features identified by computational recognition modules, including anatomical characteristics (e.g., area and shape) and patterns (e.g., correlation and neighborship) of tumor cells. The Zernike shape features might predict survival outcomes in LUAD, which was consistent with previous studies (Yu et al., 2016; Luo et al., 2017). Texture features may also be related to prognosis, such as correlation, granularity, and sum entropy. However, the prognostic power of individual image features was limited and controversial; thus, we combined image features by random forest method to provide better survival prediction. Compared to the previous researches using publicly available datasets in lung cancer (Yu et al., 2016; Luo et al., 2017; Wang et al., 2018), we extended the verification of prognostic model with four TMA validation sets, besides the internal test set. Our model successfully predicted survival results in three validation sets, which proved that the random forest model had certain robustness and generalizability across external cohorts. The prognostic ability of model was weak in validation set 1, possibly because of the heterogeneity of patients collected from different institutions. Moreover, some confounding factors that potentially affect prognosis were unknown in TMA datasets, such as smoking history, complication, and adjuvant therapy. It also suggested that our model still needed to be improved. Further training on large samples was required to adapt to the heterogeneous histopathological images of different populations.

Since the tumor progression is the result of complex biological processes, multiple molecular levels of data can describe more characteristics of tumors, which may contribute to the prognosis evaluation and therapeutic intervention (Gallo Cantafio et al., 2018). Several studies have combined histopathological image features with gene transcription data to improve survival prediction in LUAD (Zhu et al., 2016; Yu et al., 2017). The difference was that our study also involved the analysis of somatic mutation and protein expression of LUAD. Moreover, the time-dependent ROC curve was used to calculate dynamic AUC value of whole survival time (Kamarudin et al., 2017), rather than simply dividing the patients into short-term or long-term survivors. We founded that the proteomics model had higher accuracy than the models based on gene mutation or transcription, possibly because proteins were the functional executors of the cells. Proteomic analysis also showed the prognostic significance of expressed proteins and proteomics clustering in LUAD (Xu et al., 2020). Afterward, we developed and verified the prognostic models based on diverse feature integration of genomics, transcriptomics, proteomics, and histopathological images. The prognostic performance of the models using image features and single omics outperformed than the models using only one type of data. The model integrating multi-omics with image features achieved the best performance, which may contribute significantly to personalized risk stratification for LUAD patients.

There were some limitations in this study. Firstly, for genetic aberrations and subtype prediction, the small number of positive cases limited the accuracy; thus, it was necessary to expand the study sample in the future. Secondly, although we validated the prognostic model by four TMA validation sets, these validation sets were less diverse due to the TMAs processed by the same institution. The cases from TCGA and TMA datasets may exist potential biases, because representative images were more likely to be chosen. Therefore, the application of the prognostic model in actual clinical practice needed further investigation. In addition, the retrospective TMA datasets lacked genetic data; thus, the efficacy and generalizability of integrative prognostic models remained to be validated. Furthermore, the threshold of risk score was simply based on the median value, and the large-scale research would more rigorously determine the threshold to optimize patient stratification.

## Conclusion

In conclusion, our findings demonstrated that the machine learning models based on histopathological image features had great potential to predict genetic aberrations, transcriptional subtypes, and survival outcomes in patients with LUAD. In addition, the workflow of integrating histopathological image features, genomics, transcriptomics, and proteomics to develop models may improve survival prediction and benefit the precision medicine of LUAD patients.

## Data Availability Statement

The datasets presented in this study can be found in online repositories. The names of the repository/repositories and accession number(s) can be found in the article/Supplementary Material.

## Ethics Statement

The studies involving human participants were reviewed and approved by the National Human Genetic Resources Sharing Service Platform (2005DKA21300). The patients/participants provided their written informed consent to participate in this study.

## Author Contributions

XM, LC, and HZ contributed to the conception and design of the study. LC and HZ performed the data analysis, interpretation, and manuscript drafting. YX, YH, and YL provided and assessed the clinicopathological data. All authors contributed to manuscript revision and approved the submitted version.

## Conflict of Interest

The authors declare that the research was conducted in the absence of any commercial or financial relationships that could be construed as a potential conflict of interest.

## Publisher’s Note

All claims expressed in this article are solely those of the authors and do not necessarily represent those of their affiliated organizations, or those of the publisher, the editors and the reviewers. Any product that may be evaluated in this article, or claim that may be made by its manufacturer, is not guaranteed or endorsed by the publisher.

## Footnotes

1
http://www.cancerimagingarchive.net/

2
https://portal.gdc.cancer.gov/

3
http://tcpaportal.org/tcpa/

4
http://metascape.org

## Supplementary Material

Supplementary Figure 1 Examples of whole-slide histopathological images and part of selected sub-images.

Supplementary Figure 2 Kaplan-Meier analysis of groups with high-value and low-value image features.

Supplementary Figure 3 Histopathological image examples of high-risk and low-risk patients. Survival risk score was estimated by the model of histopathological image features, and then patients in the TCGA and TMA cohorts were divided into high-risk and low-risk groups by median risk score. The sub-images were processed with cell segmentation.

Supplementary Table 1 Prediction of genetic aberrations and transcriptional subtypes by machine learning algorithms in test set.

Supplementary Table 2 Hazard ratio of histopathological image features in univariate Cox analysis.

Supplementary Table 3 Gene mutation frequency in training set.

Supplementary Table 4 Differently expressed genes in groups with short or long survival.

Supplementary Table 5 Included proteins.
