# Supplementary figures and images for: Histopathological Images and Multi-Omics Integration Predict Molecular Characteristics and Survival in Lung Adenocarcinoma
Source: Front Cell Dev Biol. 2021 Oct 11;9:720110. doi: 10.3389/fcell.2021.720110 (PMC8542778; doi:10.3389/fcell.2021.720110)

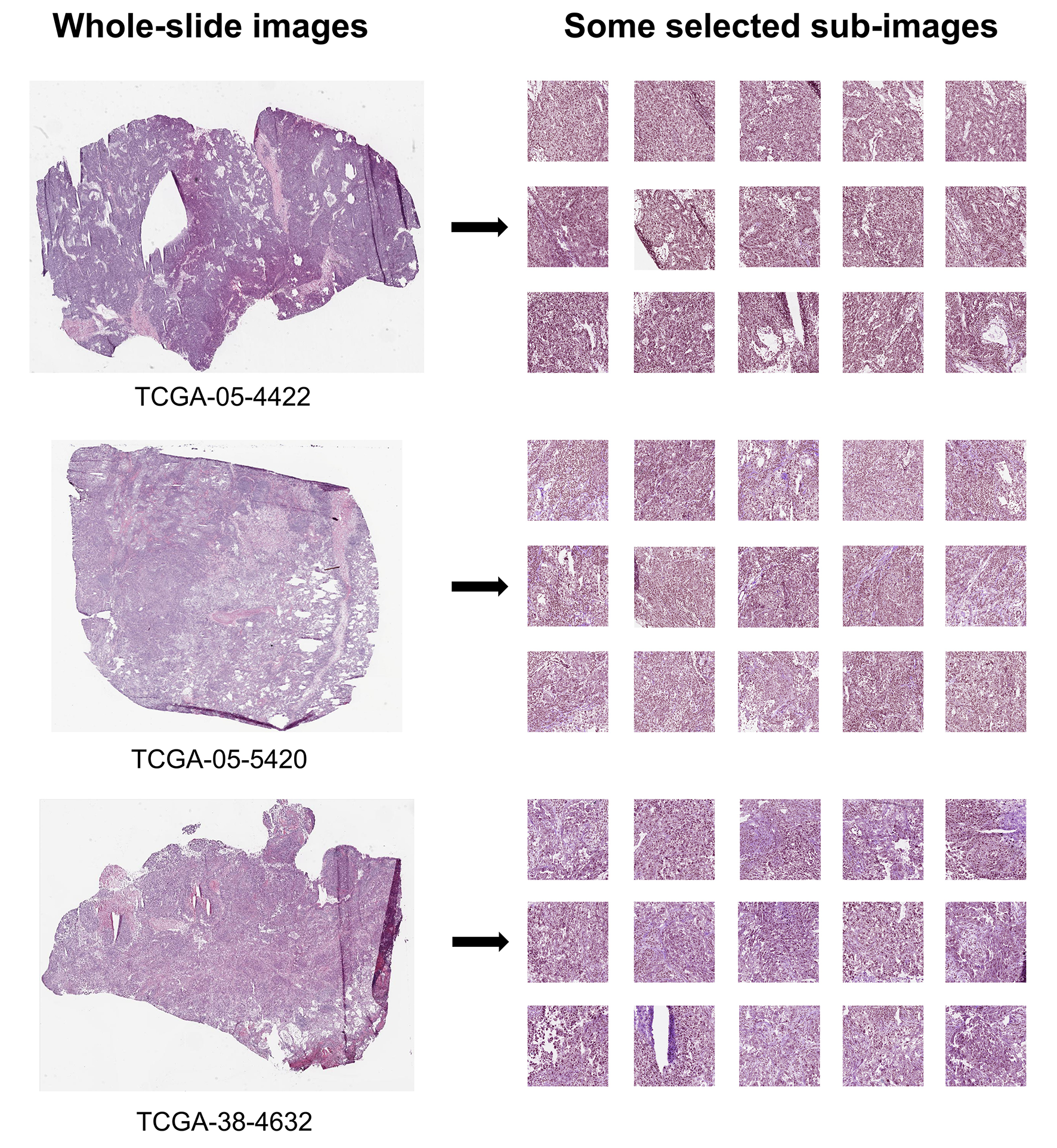

Supplement: Supplementary Figure 1 — Examples of whole-slide histopathological images and part of selected sub-images. [file Image_1.TIF]

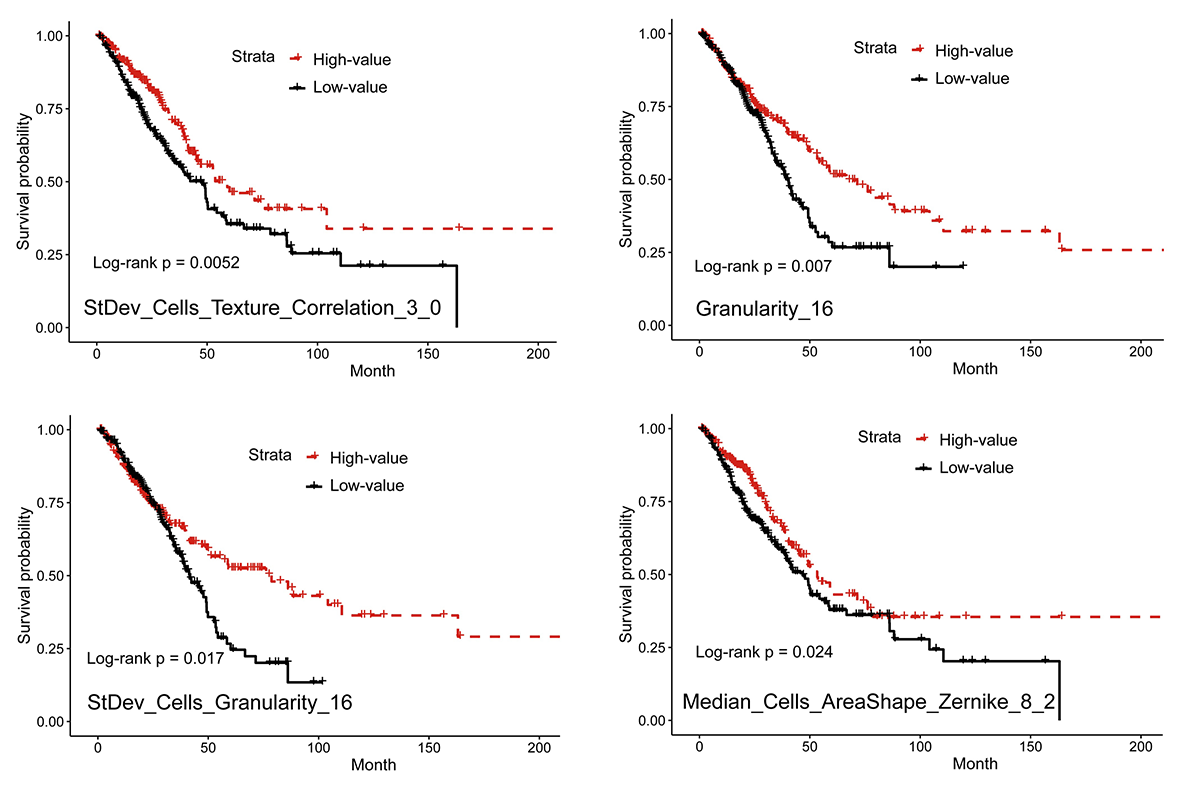

Supplement: Supplementary Figure 2 — Kaplan-Meier analysis of groups with high-value and low-value image features. [file Image_2.TIF]

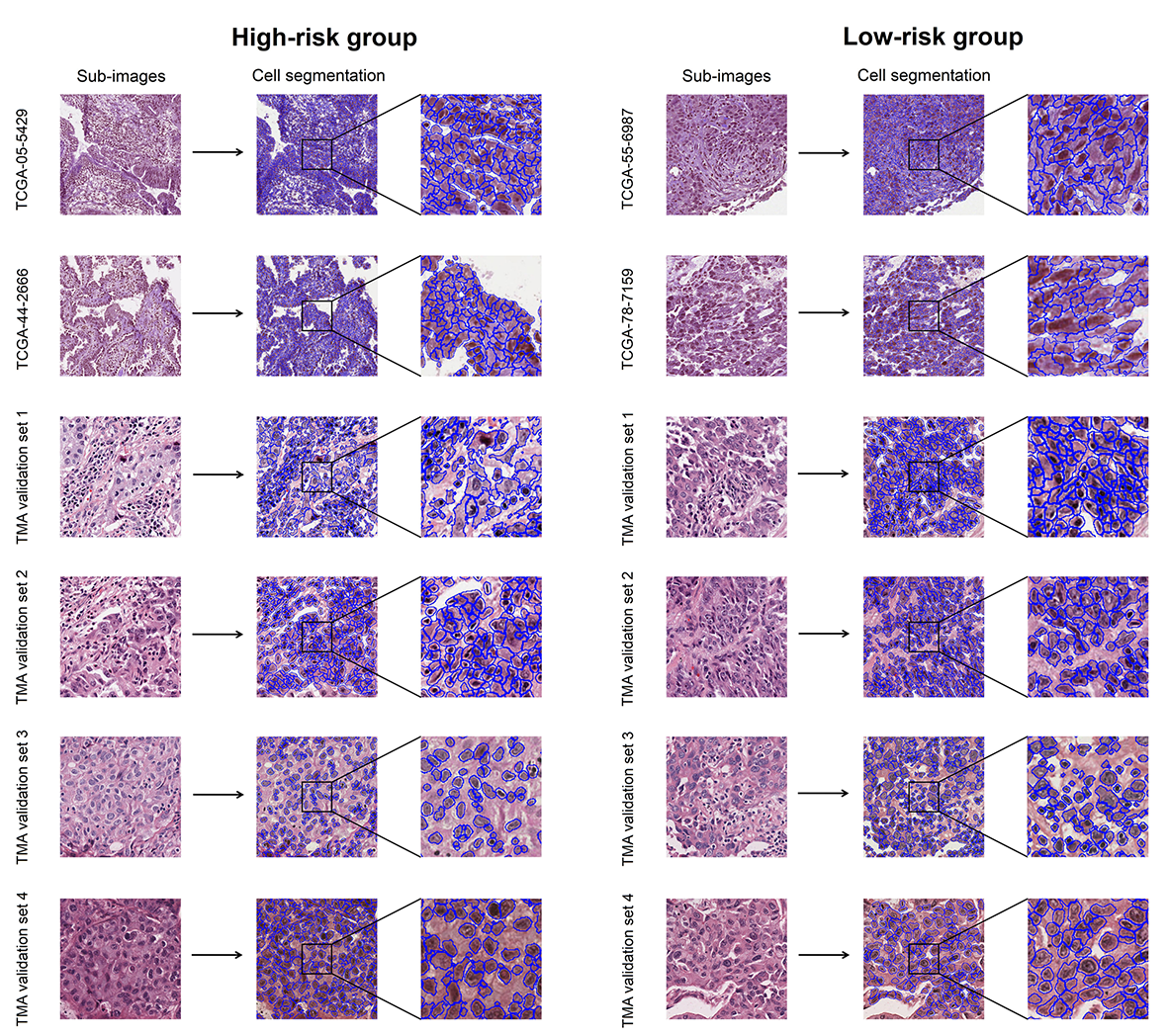

Supplement: Supplementary Figure 3 — Histopathological image examples of high-risk and low-risk patients. Survival risk score was estimated by the model of histopathological image features, and then patients in the TCGA and TMA cohorts were divided into high-risk and low-risk groups by median risk score. The sub-images were processed with cell segmentation. [file Image_3.TIF]
