# Supplementary material for: Histopathological Images and Multi-Omics Integration Predict Molecular Characteristics and Survival in Lung Adenocarcinoma
Source: Front Cell Dev Biol. 2021 Oct 11;9:720110. doi: 10.3389/fcell.2021.720110 (PMC8542778; doi:10.3389/fcell.2021.720110)
Supplement: Supplementary Table 1 — Prediction of genetic aberrations and transcriptional subtypes by machine learning algorithms in test set. [file Table_1.DOC]

**Table S1. Prediction of genetic aberrations and transcriptional subtypes by machine learning algorithms in test set.**

| **Feature**  **selection** | **Model**  **construction** | **Genetic aberration** | | | |  | **Transcriptional subtype** | | |
| --- | --- | --- | --- | --- | --- | --- | --- | --- | --- |
|  |  | **AKT** | **BRAF** | **EGFR** | **ROS1** |  | **PI** | **PP** | **TRU** |
| GBDT | RF | 0.821 | 0.771 | 0.806 | 0.802 |  | 0.907 | 0.848 | 0.843 |
|  | GBDT | 0.762 | 0.735 | 0.748 | 0.773 |  | 0.779 | 0.827 | 0.834 |
|  | AdaBoost | 0.720 | 0.784 | 0.760 | 0.731 |  | 0.810 | 0.782 | 0.810 |
|  | LR | 0.657 | 0.700 | 0.681 | 0.682 |  | 0.796 | 0.764 | 0.708 |
|  | NB | 0.661 | 0.589 | 0.595 | 0.632 |  | 0.696 | 0.724 | 0.727 |
|  | SVM | 0.622 | 0.533 | 0.500 | 0.518 |  | 0.660 | 0.742 | 0.728 |
|  | DT | 0.593 | 0.500 | 0.603 | 0.500 |  | 0.702 | 0.651 | 0.733 |
|  | KNN | 0.512 | 0.522 | 0.520 | 0.523 |  | 0.558 | 0.433 | 0.704 |
| LASSO | RF | 0.765 | 0.788 | 0.770 | 0.807 |  | 0.867 | 0.809 | 0.823 |
|  | GBDT | 0.702 | 0.738 | 0.703 | 0.792 |  | 0.786 | 0.775 | 0.833 |
|  | AdaBoost | 0.630 | 0.763 | 0.764 | 0.678 |  | 0.743 | 0.761 | 0.769 |
|  | LR | 0.616 | 0.682 | 0.638 | 0.659 |  | 0.697 | 0.771 | 0.739 |
|  | NB | 0.570 | 0.655 | 0.569 | 0.515 |  | 0.724 | 0.669 | 0.662 |
|  | SVM | 0.500 | 0.551 | 0.500 | 0.500 |  | 0.764 | 0.697 | 0.700 |
|  | DT | 0.500 | 0.630 | 0.500 | 0.500 |  | 0.640 | 0.651 | 0.687 |
|  | KNN | 0.503 | 0.554 | 0.515 | 0.517 |  | 0.534 | 0.518 | 0.567 |
| RF | RF | 0.879 | 0.847 | 0.855 | 0.848 |  | 0.897 | 0.861 | 0.894 |
|  | GBDT | 0.813 | 0.730 | 0.802 | 0.859 |  | 0.830 | 0.802 | 0.812 |
|  | AdaBoost | 0.733 | 0.744 | 0.719 | 0.729 |  | 0.795 | 0.813 | 0.795 |
|  | LR | 0.690 | 0.618 | 0.694 | 0.707 |  | 0.816 | 0.665 | 0.762 |
|  | NB | 0.610 | 0.605 | 0.619 | 0.590 |  | 0.683 | 0.647 | 0.699 |
|  | SVM | 0.560 | 0.500 | 0.513 | 0.500 |  | 0.684 | 0.753 | 0.709 |
|  | DT | 0.547 | 0.500 | 0.614 | 0.500 |  | 0.690 | 0.651 | 0.710 |
|  | KNN | 0.512 | 0.513 | 0.526 | 0.543 |  | 0.510 | 0.525 | 0.551 |
| XGBoost | RF | 0.897 | 0.830 | 0.843 | 0.855 |  | 0.948 | 0.828 | 0.889 |
|  | GBDT | 0.831 | 0.811 | 0.775 | 0.803 |  | 0.906 | 0.725 | 0.879 |
|  | AdaBoost | 0.750 | 0.785 | 0.734 | 0.732 |  | 0.829 | 0.695 | 0.885 |
|  | LR | 0.770 | 0.728 | 0.698 | 0.774 |  | 0.802 | 0.767 | 0.740 |
|  | NB | 0.656 | 0.697 | 0.611 | 0.642 |  | 0.715 | 0.667 | 0.780 |
|  | SVM | 0.604 | 0.499 | 0.526 | 0.517 |  | 0.673 | 0.755 | 0.784 |
|  | DT | 0.547 | 0.500 | 0.580 | 0.500 |  | 0.729 | 0.665 | 0.733 |
|  | KNN | 0.543 | 0.588 | 0.516 | 0.486 |  | 0.559 | 0.652 | 0.565 |

Abbreviations: ALK, anaplastic lymphoma kinase; BRAF, v-Raf murine sarcoma viral oncogene homolog B1; EGFR, epidermal growth factor receptor ROS; ROS1, ROS proto-oncogene 1 receptor tyrosine kinase; PI, proximal-inflammatory; PP, proximal-proliferative; TRU, terminal respiratory unit; GBDT, gradient boosting decision tree; LASSO, least absolute shrinkage and selection operator; RF, random forest; XGBoost, extreme gradient boosting; AdaBoost, adaptive boosting; LR, logistic regression; NB, naive Bayes; SVM, support vector machine; DT, decision tree; KNN, K-nearest neighbor.
